# Multi-omics analysis of gut microbiota and metabolites reveals contrasting profiles in domestic pigs and wild boars across urban environments

**DOI:** 10.3389/fmicb.2024.1450306

**Published:** 2024-08-13

**Authors:** Jingjing Ding, Xinyuan Cui, Xuan Wang, Feifei Zhai, Lei Wang, Lifeng Zhu

**Affiliations:** ^1^Jiangsu Academy of Forestry, Nanjing, China; ^2^Jiangsu Yangzhou Urban Forest Ecosystem National Observation and Research Station, Yangzhou, China; ^3^College of Pharmacy, Nanjing University of Chinese Medicine, Nanjing, China; ^4^Jiangsu Wildlife Protection Station, Nanjing, China

**Keywords:** wild boar, gut microbiome, metablome, ARGs, environmental contamination

## Abstract

The gut microbiota plays a crucial role in host health and metabolism. This study explores the differences in gut microbiota and metabolites between domestic pigs (DP) and wild boars (WB) in urban environments. We analyzed gut microbial composition, metabolic profiles, virome composition, antibiotic resistance genes (ARGs), and human pathogenic bacteria (HPB) in both DP and WB. Our results revealed that DP exhibited a higher Firmicutes/Bacteroidetes ratio and were enriched in bacterial genera associated with domestication and modern feeding practices. Metabolomic analysis showed distinct profiles, with WB significantly enriched in the Pantothenate and CoA biosynthesis pathway, highlighting dietary and environmental influences on host metabolism. Additionally, DP had a distinct gut virome composition, particularly enriched in lytic phages of the Chaseviridae family. ARG analysis indicated a higher abundance of tetracycline resistance genes in DP, likely due to antibiotic use in pig farms. Furthermore, variations in HPB composition underscored potential health risks associated with contact with pig feces. These findings provide valuable insights into the microbial ecology of domestic pigs and wild boars, emphasizing the importance of these comparisons in identifying zoonotic pathogen transmission pathways and managing antibiotic resistance. Continued research in this area is essential for developing effective strategies to mitigate public health risks and promote sustainable livestock management practices.

## 1 Introduction

The gut microbiota, consisting of trillions of microorganisms like bacteria, viruses, fungi, and protozoa, forms a complex symbiotic ecosystem with the host (Barko et al., 2018). This symbiotic relationship is pivotal for host health, immunity, and disease resistance (Rooks and Garrett, 2016; Afzaal et al., 2022). Recent studies highlight the gut microbiota’s significant role in both gastrointestinal and systemic health (Caputi et al., 2021). Furthermore, metabolites are critical regulatory factors and essential components for maintaining host growth, development, and health (Liu et al., 2022; Zhang et al., 2023). Metabolites produced by the gut microbiota provide necessary energy and promote physiological activities through compounds like short-chain fatty acids and secondary bile acids (Rowland et al., 2018; Rahman et al., 2023).

Domestic pigs (*Sus domesticus*, DP) and wild boars (*Sus scrofa*, WB), represent two distinct populations with contrasting ecological niches, diets, and lifestyles. Centuries of selective breeding and domestication have led to significant genetic and phenotypic changes compared to their wild ancestors, wild boars (Larson et al., 2005; Yang et al., 2018, 2020). Studies show that DP have developed a *lactobacillus*-dominated enterotype due to domestication and human feeding mechanisms, while WB retain a Bifidobacterium-dominated microbiome (Ushida et al., 2016; Yang et al., 2020; Kuthyar et al., 2023; Hu et al., 2024). Urbanization reduced the buffer zone between WB habitats and cities, increasing human-wildlife interactions and conflicts (Lamichhane et al., 2019; Li et al., 2022; Simkin et al., 2022). Furthermore, the absence of natural predators for WB in urban areas may contribute to a steady increase in their populations (Conejero et al., 2019; Castillo-Contreras et al., 2021; Zhou et al., 2023). Additionally, urban environments provide new ecological pressures and dietary patterns for WB, potentially altering their gut microbiota (Stillfried et al., 2017; Castillo-Contreras et al., 2021; Davidson et al., 2022). In contrast, DP are typically raised in controlled environments with formulated diets and management practices, including antibiotic use and vaccination (Maes et al., 2020; Rodrigues et al., 2022). WB inhabit diverse nature ecosystems, primarily consuming plants but also preying on invertebrates, small mammals, and carrion opportunistically (Stillfried et al., 2017; Ruf et al., 2021). Consequently, these changes in diet and habitat may lead to significant alterations in the gut microbiota of urban wild boars.

Furthermore, the proximity of wild boars to human settlements raises concerns regarding public health and safety. WB act as reservoirs for a plethora of human pathogenic bacteria (HPB) and antibiotic-resistant genes (ARGs), posing a significant risk of disease transmission to humans and domestic animals (Meng et al., 2009; Fredriksson-Ahomaa, 2019; Dias et al., 2022). Increased interactions in urban areas facilitate the exchange of infectious viruses, ARGs, and HPB, potentially leading to zoonotic disease outbreaks (Wierucka et al., 2023). Identified viruses in WB include hepatitis E virus (HEV), influenza A virus (IAV), and porcine reproductive and respiratory syndrome virus (PRRSV) (Chen et al., 2018; Liang et al., 2019). Additionally, key ARGs in WB confer resistance to beta-lactams, tetracyclines, fluoroquinolones, aminoglycosides, and sulfonamides, often originating from agricultural runoff or human waste (Fredriksson-Ahomaa, 2019; Torres et al., 2020; Selmi et al., 2022). The spread of ARGs, facilitated by horizontal gene transfer, complicates treatment and poses a threat to human health (Dias et al., 2022). Additionally, wild boars carry various HPB, including *Salmonella*, *Campylobacter*, *Escherichia coli*, and *Yersinia enterocolitica*, which can cause gastrointestinal illnesses in humans through contaminated food, water, or direct contact with WBs or their feces (Meng et al., 2009; Ali and Alsayeqh, 2022). Urbanization-driven increases in WB populations heighten the risk of pathogen transmission through closer human contact and environmental contamination.

In summary, urbanization and wild boar encroachment into urban areas pose significant ecological and public health challenges (Basak et al., 2022). Effective management strategies are needed to address human-wildlife conflicts and disease transmission risks. Analyzing the gut microbiota of domestic pigs and wild boars in urban areas is crucial for assessing zoonotic disease transmission and antibiotic resistance. Continued surveillance and research efforts are essential to monitor infectious viruses, ARGs, and human pathogenic bacteria, helping to mitigate public health risks.

## 2 Materials and methods

### 2.1 Animals and sample collection

In this study, fecal samples were collected from domestic pigs (DP) and wild boars (WB), comprising 8 domestic pigs and 28 wild boars. Further details on the sample information were provided in Supplementary Tables S1–S3. All animals were sourced from Nanjing City, Jiangsu Province, China. The domestic pig samples included 2 DP samples from a pig farm in Pukou District and 6 DP samples from a pig farm in Jiangning District. Fecal samples from domestic pigs were obtained directly from their anus, placed in sterile sampling bottles, transported to the laboratory at 4°C, and stored at −80°C for long-term preservation. Regarding wild boar fecal samples, 4 WB samples were collected from a pig farm in Pukou District, 4 WB samples from a pig farm in Jiangning District, and 20 WB samples from a pig farm in Xuanwu District. For wild boars in their natural habitat, precautions were taken to minimize soil contamination. Fresh feces were gathered from the ground using sterile tweezers, transferred to sterile sampling bottles, transported to the laboratory at 4°C, and stored at −80°C for long-term storage.

### 2.2 DNA extraction and sequencing of full-length 16S rRNA and metagenomic samples

DNA was extracted using the QIAamp DNA Stool Mini Kit according to the manufacturer’s instructions. The concentration of DNA was determined by 1% agarose gel electrophoresis (DNA concentration ≥ 20 ng/μl). For full-length 16S rRNA analysis, the universal primer set 27F (AGRGTTYGATYMTGGCTCAG) and 1492R (RGYTACCTTGTTACGACTT) was used to amplify the full-length 16S rRNA gene from genomic DNA. PCR products were detected by 2% agarose gel electrophoresis. Gel extraction of PCR products was performed using the AxyPrepDNA Gel Extraction Kit (AXYGEN), followed by elution with Tris–HCl buffer. The recovered PCR products were further analyzed by 2% agarose gel electrophoresis. For metagenomic analysis, post-DNA extraction, DNA was fragmented into approximately 300 bp fragments using a Covaris M220 sonicator. “Y”-shaped adapters were ligated to both ends of the DNA fragments, followed by magnetic bead selection to remove self-ligated fragments. Subsequently, PCR amplification was performed to construct the library. Library construction was carried out using the TruSeq™ DNA Sample Prep Kit, with library quantification via qPCR ensuring a library concentration greater than 3 nM. Next, bridge PCR amplification was conducted using the cBot Truseq PE Cluster Kit v3-cBot-HS. Following library qualification, high-throughput sequencing was performed on the Illumina HiSeq platform for full-length 16S rRNA sequencing and metagenomic sequencing. After sequencing data acquisition, sequences shorter than 500 bp were initially removed from the raw data. Subsequently, a series of processing steps including sequence correction, dereplication, and adapter trimming were carried out as in previous methods (Cui et al., 2023; Dai et al., 2023) to obtain high-quality representative sequences.

### 2.3 Bioinformatics analysis of 16S rRNA data

OTU clustering analysis was conducted using Usearch software (version 10, http://drive5.com/uparse/) (Edgar, 2013). Initially, non-redundant sequences were extracted from the optimized sequences, and singleton sequences were eliminated. Subsequently, non-redundant sequences were clustered into OTUs at 97% similarity, while chimeras were simultaneously excluded, resulting in representative sequences for each OTU. These optimized sequences were then aligned to the representative sequences of OTUs, and sequences showing similarity greater than 97% to the representative sequences were filtered out to generate the requisite OTU table. Finally, the OTU table underwent rarefaction to ensure uniform sequencing depth across samples. Subsequently, the representative sequences of OTUs were processed using the uclust software (Edgar, 2010) to enumerate the microbial community composition of wild boar and domestic pig samples at different taxonomic levels (including domain, kingdom, phylum, class, order, family, genus, and species).

Principal Coordinate Analysis (PCoA) and Non-metric Multidimensional Scaling (NMDS) analyses, utilizing Bray–Curtis dissimilarity, were conducted to visually represent the similarities and dissimilarities within and between sample groups. A Venn diagram was utilized to elucidate the shared and unique microbial taxa between the two groups, namely the WB and DP groups. This graphical representation provides a clear depiction of the overlap and distinctiveness of microbial communities. To further explore the differences in microbial composition between the groups, heatmaps were employed. Heatmaps provide a concise visual summary of microbial abundance patterns across samples, facilitating the identification of taxa exhibiting differential abundance between groups. This analytical tool aids in pinpointing key microbial signatures associated with specific sample conditions. Additionally, Linear discriminant analysis Effect Size (LEfSe) (Chang et al., 2022), a widely recognized tool for biomarker discovery, was employed. Initially, non-parametric factorial Kruskal-Wallis and Wilcoxon rank-sum tests were conducted to identify features with significantly different abundances among the samples. Subsequently, the Linear Discriminant Analysis (LDA) within LEfSe was utilized to assess the impact of each component’s abundance on the observed differences. The results were visualized using bar plots, with features having an LDA score > 3.0 and *p* < 0.05 considered significant biomarkers.

### 2.4 Bioinformatics analysis of metagenomic data

The BLAST (version 2.2.28+, http://blast.ncbi.nlm.nih.gov/Blast.cgi) was utilized to align the metagenomic gene sequences and annotate them using multiple databases. The Seed database was employed for annotating viral components, which encompassed extensive viral-related information. For annotating antibiotic resistance genes, data from the Antibiotic Resistance Gene Database (ARDB), the Comprehensive Antibiotic Resistance Database (CARD), and non-redundant data from the NCBI database were integrated to construct the SARG dataset, ensuring comprehensive annotation of antibiotic resistance genes. Furthermore, the NCBI and VFDB databases were utilized for annotating Human Pathogenic Bacteria (HPB), to obtain detailed information related to human pathogenic bacteria. Finally, by analyzing the alignment results, the annotation outcomes were associated with the original sequences to identify features relevant to viral components, antibiotic resistance genes, and HPB, thereby further exploring the biological significance of these features within the microbial community. Based on the annotated classification information of the metagenome, stacked bar charts were employed to illustrate the composition and relative abundances of viral communities, antibiotic resistance genes (ARGs), and Human Pathogenic Bacteria (HPB) within each sample group. Furthermore, the Stamp (Shaufi et al., 2015) was utilized for differential analysis to determine their significant differences across different groupings (confidence interval is 95%, *p* < 0.05). Additionally, heatmap visualization was employed to analyze the relative abundances of these differentially represented species, allowing for a deeper understanding of the microbial community compositional differences between the two sample groups.

### 2.5 Metabolome analysis

Fecal samples from domestic pigs (DP) and wild boars (WB) were also utilized in the metabolome experiment, which was conducted by Shanghai Mingke Biotechnology (Hangzhou) Co., Ltd. The detailed Metabolome protocol was provided in the Supplementary material. To ensure the reliability and reproducibility of metabolomic data and highlight their biological significance, we conducted a series of preparations and treatments. Initially, we employed deviation and missing value filtrations to identify and eliminate noise, outliers, and missing data, thereby retaining high-quality data. Specifically, we computed the relative standard deviation (RSD) for each peak and applied appropriate thresholds based on experimental conditions and data characteristics for filtration. Regarding missing value filtration, we retained only high-quality data and utilized imputation methods to fill missing values, ensuring data integrity and stability. For data standardization, we selected suitable internal standards for sample normalization, mitigating technical variability among different samples. We established precursor tolerances of 5 ppm, product tolerances of 10 ppm, and a 5% product threshold for compound identification. Compound identification relied on mass-to-charge ratio (M/z), secondary fragments, and isotopic distribution, employing databases including the Human Metabolome Database (HMDB) and Kyoto Encyclopedia of Genes and Genomes (KEGG) for metabolite annotation and qualitative analysis. Extracted data underwent further processing, wherein peaks with missing values exceeding 50% in groups and compounds scoring below 36 points were removed.

Principal Component Analysis (PCA) and Partial Least Squares Discriminant Analysis (PLS-DA) were employed to evaluate the overall distributional trends and the extent of differences between samples across different groups. Utilizing the Pheatmap package in R (Li et al., 2023), we constructed heatmaps with Z-score conversion to visually depict the concentration of enriched metabolites, thereby emphasizing the biological significance of the data and variations in metabolites among distinct samples. Subsequently, we extracted metabolite compositions from each database and utilized MetaboSignal (Rodriguez-Martinez et al., 2017) to generate enrichment information for KEGG pathways based on metabolite compositions. Bacterial genera displaying significant differences among groups of microbial samples corresponding to metabolome samples were identified using the Wilcoxon rank-sum test. Spearman correlation analysis was conducted to explore the associations between bacterial genera and metabolites employing the R package psych. Correlations were deemed significant at a threshold of *p* < 0.05 (Hu et al., 2022). Finally, a correlation heatmap was generated utilizing the R package pheatmap.

## 3 Results and discussions

### 3.1 Gut microbial composition differences between DP and WB

**Figure 1:**
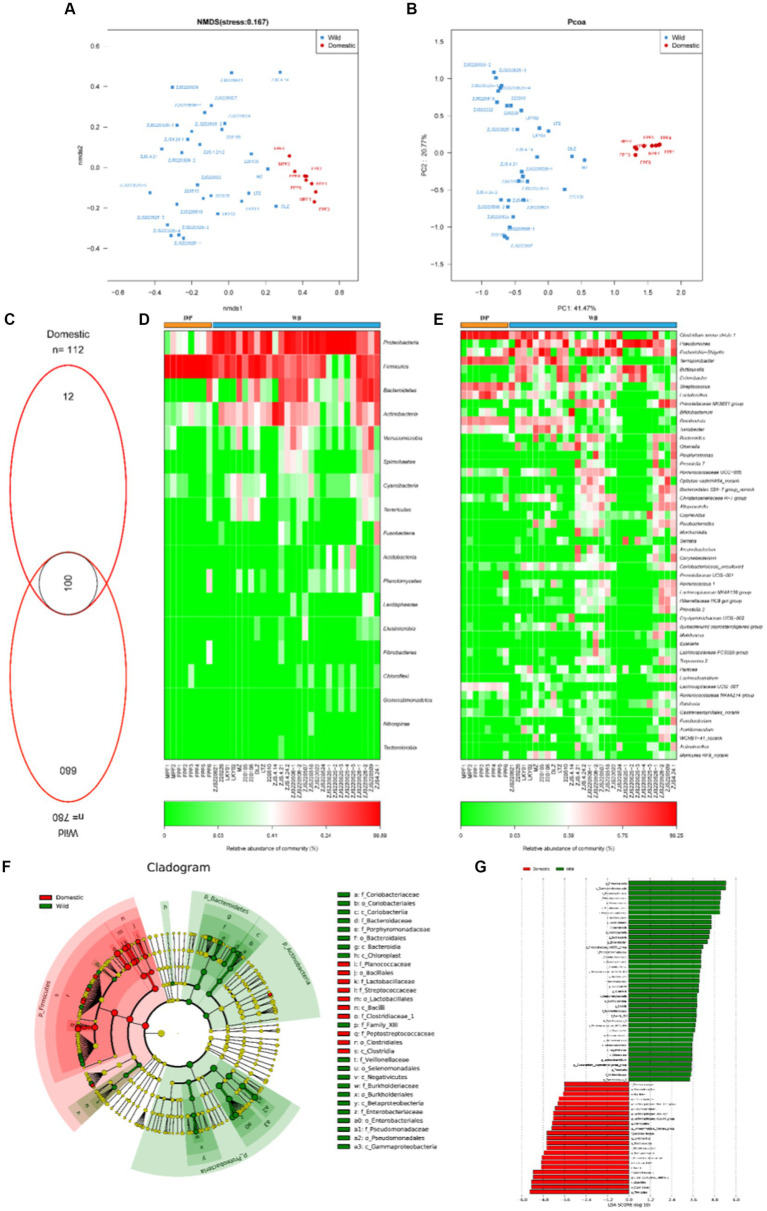
The composition of gut microbiome in DP and WB. **(A)** NMDS analysis based on Bray–curtis distance; **(B)** PCoA analysis based on Bray–curtis distance; **(C)** Composition of OTUs in DP and WB; **(D)** The relative abundance of the dominant phylum in DP and WB; **(E)** The relative abundance of the dominant genus in DP and WB; **(F)** LEfSe (Linear discriminant analysis Effect Size) was used to determine the significant difference in the abundance of gut microbiomes between DP and WB; **(G)** Histogram of LDA values of gut differential species of DP and WP (LDA value > 3.0, *p* < 0.05).

We analyzed 16S rRNA gene data from 36 samples (8 domestic pigs and 28 wild boars) (Supplementary Table S1). NMDS (Figure 1A) and PCoA (Figure 1B) analyses showed clearly separated clusters of DP and WB. As shown in Figure 1C, DP and WB shared 100 OTUs of gut microbiota. DP had fewer unique gut microbiota (12 OTUs) compared to WB (680 OTUs). The main phyla in the gut microbiome of wild boars included Proteobacteria, Firmicutes, Bacteroidetes, and Actinobacteria, while Firmicutes was overwhelmingly dominant in domestic pigs (Figure 1D). Compared to Firmicutes, Bacteroidetes has fewer genes for enzymes involved in carbohydrate and lipid metabolism, which play an important role in weight gain (Stephens et al., 2018). It has been reported that the gut microbiota of obese individuals typically exhibited a higher Firmicutes/Bacteroidetes (F/B) ratio compared to normal-weight individuals. Therefore, the F/B ratio frequently serves as a hallmark of obesity in humans and animals (Magne et al., 2020). In this study, it was observed that the F/B ratio of DP (F/B = 65.54) was remarkably higher than that of WB (F/B = 2.48), which may be more in line with the realistic demands for weight gain and livestock breeding efficiency in the pig farming industry. Additionaly, at the genus level (Figure 1E), DP contained more *Clostridium sensu stricto 1*, *Terrisporobacter*, *Streptococcus*, and *Lactobacillus*, while *Pseudomonas*, *Escherichia-Shigella*, *Buttiauxella*, and *Enterobacter* were more abundant WB. Previous studies have shown that domestication and modern feeding practices result in a gut microbiota dominated by *Lactobacillus* in domestic pigs (Ushida et al., 2016; Yang et al., 2020). Dietary factors are highly probable to be an important contributor to the variability of the gut microbiome in domestic pigs and wild boars.

Similarly, LEfSe (Linear discriminant analysis effect size) analysis also revealed the significant differences in microbial species between DP and WB (LDA score > 3.0, *p* < 0.05) (Figures 1F,G). A total of 64 significantly differential microbial taxa were identified in DP and WB (Figure 1G). Among those, Enterobacteriaceae, Pseudomonadaceae, Porphyromonadaceae, Coriobacteriaceae, Bacteroidaceae, Burkholderiaceae, Family_XIII, and Veillonellaceae were identified as WB enriched gut bacteria, whereas Clostridiaceae_1, Peptostreptococcaceae, Streptococcaceae, Lactobacillaceae, and Planococcaceae were significantly enriched within DP. Prior research has shown a dissimilarity in gut microbiota among wild boars, commercial pigs, and domestic pigs. Commercial pigs displayed higher levels of Streptococcaceae and Lactobacillaceae, whereas Ruminococcaceae and Prevotellaceae were more abundant in wild boars than in the other groups (Yang et al., 2020). In this study, some gut bacteria genus belonged to Ruminococcaceae and Prevotellaceae, include *Ruminococcus_1, Ruminococcus_2, Ruminococcaceae_UCG_013, Ruminococcaceae_UCG_014, Eubacterium_coprostanoligenes_group*, and *Prevotellaceae_NK3B31_group* were also observed to be significant enriched in WB (Figure 1G).

### 3.2 Dissimilarity of metabolic profiles in DP and WB

**Figure 2:**
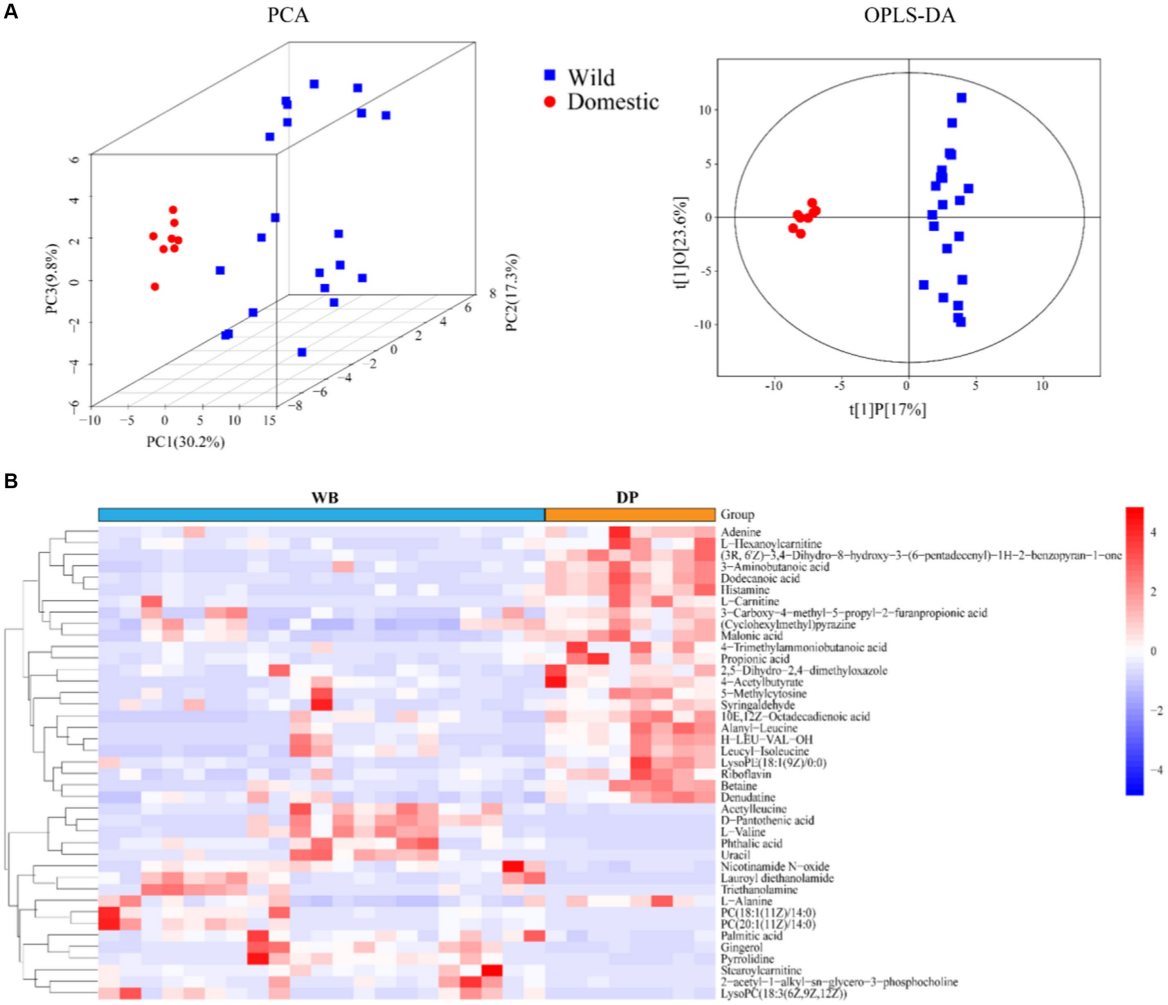
The metabolite profiles of DP and WB. **(A)** PCA (based on Bray–curtis distance) and OPLS-DA score of DP and WB; **(B)** Heat map of 41 metabolites with significant differences between DP and WB, red and blue represent higher and lower concentrations of metabolites in DP and WB, respectively.

A non-targeted metabolomic analysis was performed to examine the metabolite profiles in fecal samples from domestic pigs and wild boars. A total of 164 metabolites were identified, belonging to 6 super classes (Supplementary Figure S1), including Benzenoids (4.88%), Lipids and lipid−like molecules (34.15%), Organic acids and derivatives (19.51%), Organic nitrogen compounds (7.32%), Organoheterocyclic compounds (21.95%), and Others (12.2%). The PCA score plot and OPLS-DA model demonstrated clear separation between DP and WB (Figure 2A), with a total of 41 differential metabolites identified (Figure 2B). Among these, it was observed that Adenine, L-Hexanoylcarnitine, (3R, 6’Z)-3,4-Dihydro-8-hydroxy-3-(6-pentadecenyl)-1H-2-benzopyran-1-one, 3-Aminobutanoic acid, Dodecanoic acid, Histamine, L-Carnitine, 3-Carboxy-4-methyl-5-propyl-2-furanpropionic acid, (Cyclohexylmethyl)pyrazine, Malonic acid, 4-Trimethylammoniobutanoic acid, Propionic acid, 2,5-Dihydro-2,4-dimethyloxazole, 4-Acetylbutyrate, 5-Methylcytosine, Syringaldehyde, 10E,12Z-Octadecadienoic acid, Alanyl-Leucine, H-LEU-VAL-OH, Leucyl-Isoleucine, LysoPE (18:1 (9Z)/0:0), Riboflavin, Betaine, Denudatine, and L-Alanine were more abundant in DP, whereas Acetylleucine, D-Pantothenic acid, L-Valine, Phthalic acid, Uracil, Nicotinamide N-oxide, Lauroyl diethanolamide, Triethanolamine, PC (18:1 (11Z)/14:0), PC (20:1 (11Z)/14:0), Palmitic acid, Gingerol, Pyrrolidine, Stearoylcarnitine, 2-acetyl-1-alkyl-sn-glycero-3-phosphocholine, and LysoPC (18:3 (6Z,9Z,12Z)) were more predominant in WB.

**Figure 3:**
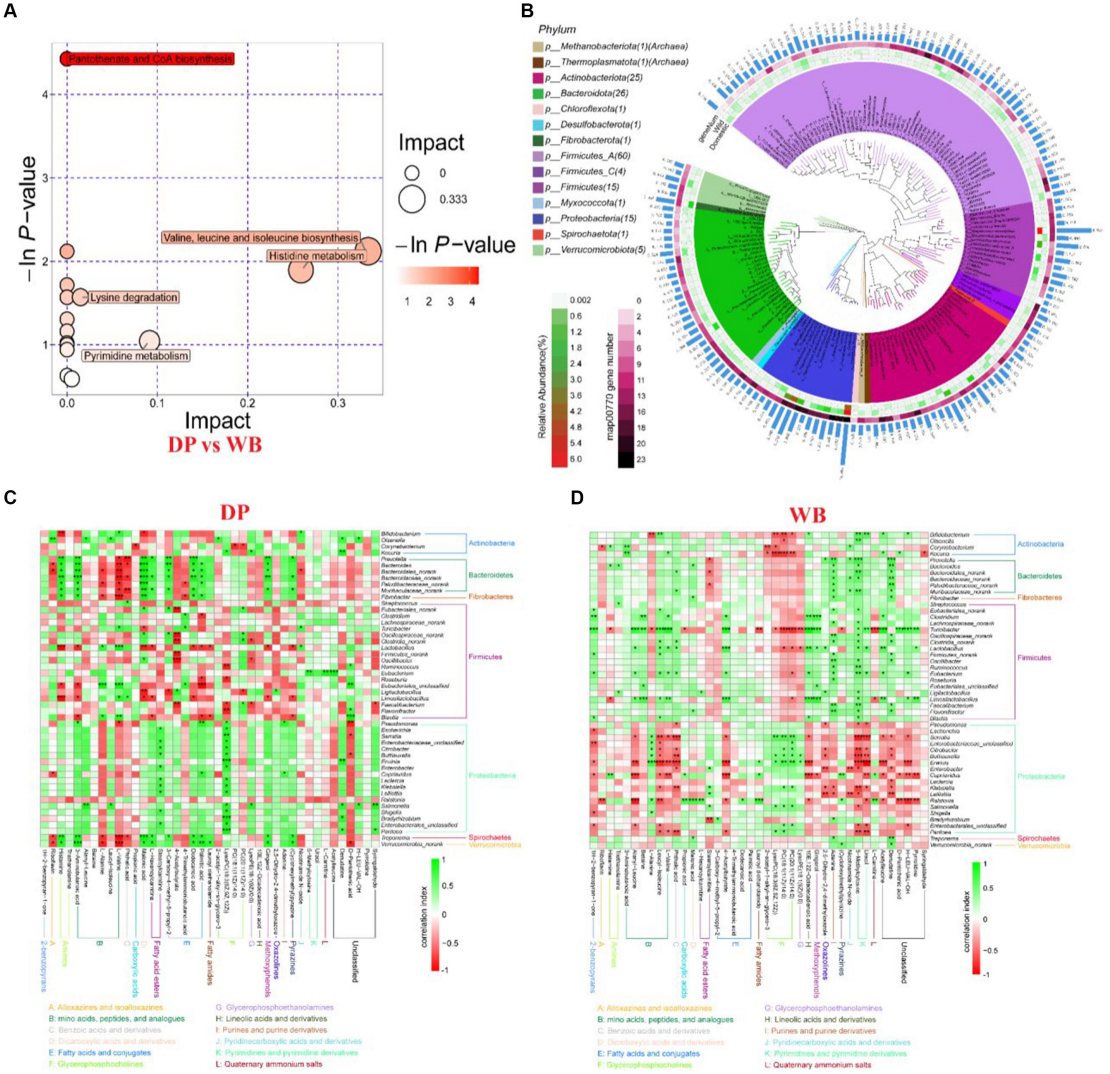
Metabolism pathway analysis. **(A)** Metabolism pathway enrichment analysis between DP and WB, the size and color of the bubble indicate pathway enrichment and impact values; **(B)** Phylogenetic analysis of MAGs, the panels in the center circle display the maximum-likelihood trees created using the MAGs. The outer circle heatmap shows the relative abundance of each bin (MAG) in DP and WB, as well as the gene numbers in map00770 (Pantothenate and CoA biosynthesis); **(C)** Correlation analysis of gut microbiota and metabolites in DP; **(D)** Correlation analysis of gut microbiota and metabolites in WB. The shade of color indicates the magnitude of the correlation coefficient, that green and red indicate positive and negative correlations, respectively. Significant correlations are indicated by black stars: *p < 0.05, **p < 0.01, ***p < 0.001.

The pathway enrichment analysis of differential fecal metabolites between DP and WB (Figure 3A) revealed a significant enrichment in the Pantothenate and CoA biosynthesis pathway (*p* = 0.011942, impact value = 0). Compared to DP, WB harbor a higher gene number associated with Pantothenate and CoA biosynthesis (KEGG Pathway: map00770) (Figure 3B), particularly within the dominant Proteobacteria (including *Citrobacter, Erwinia, Leclercia, Pseudomonas_E sp005233515, Pseudomonas_Eazotoformans_A, Serratia liquefaciens, Erwinia billingiae, Enterobacter ludwigii, Escherichia flexneri*) in WB. Pantothenate (also known as vitamin B5) is an essential vitamin precursor required for the synthesis of coenzyme A (CoA), a vital molecule involved in various metabolic pathways (de Villiers et al., 2013). Pantothenate and CoA biosynthesis plays a crucial role in various biochemical reactions, including fatty acid synthesis, amino acid metabolism, and the citric acid cycle (He et al., 2019; Naquet et al., 2020; de Vries et al., 2021). In this study, D-Pantothenic acid, L-Valine, and Uracil significantly enriched in WB were all associated with the Pantothenate and CoA biosynthesis pathway (Wu et al., 2018). L-Valine can synergistically regulate the Pantothenate and CoA biosynthesis pathway with pantothenic acid (Schicho et al., 2012; Bai et al., 2022). 48-h fasting in pigs has been demonstrated to influence Pantothenate and CoA biosynthesis by significantly enhancing pantothenate metabolism (Liu et al., 2018). Additionally, other studies indicate that heat stress (HS) (He et al., 2019; Srikanth et al., 2020) and infection with classical swine fever virus (CSFV) (Liao et al., 2023) also exert significant effects on the Pantothenate and CoA biosynthesis pathway in pigs.

Notably, it was observed in this study that the Pantothenate and CoA biosynthesis pathway, the main differential metabolites were L-Valine and Uracil, with significantly higher expression levels in WB compared to DP (Supplementary Table S2; Supplementary Figures S2, S3). Further exploration of the association between gut microbiota and host metabolism revealed that, in comparison to DP (Figure 3C), certain bacterial taxa in WB (Figure 3D) exhibited significant positive correlations with L-Valine and Uracil. For instance, Turicibacter (*p* < 0.001), Kocuria (*p* < 0.05), Eubacterium (*p* < 0.05) showed significant positive correlations with L-Valine, while Bifidobacterium (*p* < 0.01), Turicibacter (*p* < 0.01), Eubacterium (*p* < 0.05) exhibited significant positive correlations with Uracil. Some evidence confirmed that the typical characteristic of gut microbiota in wild boars, compared to domestic pigs, is the high abundance of *Bifidobacterium* (Ushida et al., 2016; Patil et al., 2020; Yang et al., 2020). Moreover, it has been suggested that a LF (low fat and high fiber) diet is more effective in increasing the levels of *Bifidobacterium* in the pig gut than a HF (high fat and low fiber) diet (Heinritz et al., 2016). In order to improve the meat quality and farming efficiency of domestic pigs, protein feed is commonly added to their daily diet (Déru et al., 2022). While the diet of domestic pigs usually comprises consistent protein sources and low fiber content, wild boars predominantly consume high-fiber plants in their natural habitat, notwithstanding their occasional consumption of animal materials like small vertebrates, invertebrates, and carrion (Stillfried et al., 2017; Vedel et al., 2023). Therefore, we hypothesize that the differences in the metabolic profiles of domestic pigs and wild boars may partly stem from differences in their gut microbiota composition driven by dietary discrepancies, with the latter playing a role in subsequently influencing the metabolic profiles.

### 3.3 Gut virome composition variations in DP and WB

**Figure 4:**
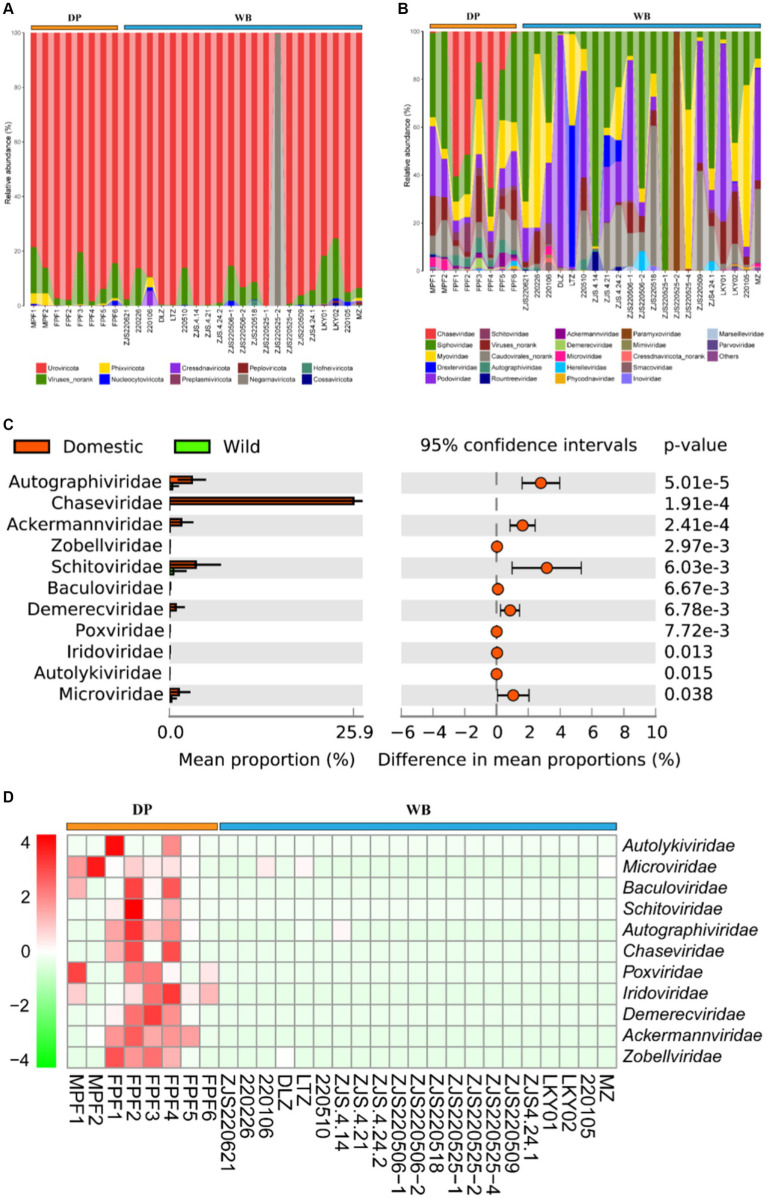
Composition and dissimilarity of gut virome between DP and WB. **(A)** The relative abundance of gut virome (phylum level) in DP and WB; **(B)** The relative abundance of gut virome (genus level) in DP and WB; **(C)** Significantly different gut virome composition between DP and WB; **(D)** Heat map of gut virome with significant differences between DP and WB, red and green represent higher and lower concentrations of metabolites in DP and WB, respectively.

Metagenomic studies revealed that Uroviricota dominates the gut virome of DP and WB (Figures 4A, B). The most abundant viral families in DP were Chaseviridae (50.00%), Schitoviridae (15.79%), Autographiviridae (9.43%), and Schitoviridae (7.17%). In WB, the top four viral families were Drexlerviridae (28.88%), Ackermannviridae (23.07%), Adenoviridae (22.16%), and Alloherpesviridae (20.41%). Stamp differential analysis reveals significant differences between DP and WB for 11 viruses (at the family level) (Figures 4C, D), including Ackermannviridae, Autographiviridae, Autolykiviridae, Baculoviridae, Chaseviridae, Demerecviridae, Iridoviridae, Microviridae, Poxviridae, Schitoviridae, and Zobellviridae. All these viruses exhibit significantly higher relative abundance in DP compared to WB, particularly Chaseviridae.

Metagenomic studies revealed that Uroviricota dominates in the gut of DP and WB (Figure 4A). At the family level, the most abundant viral compositions in DP were Chaseviridae (50.00%), followed by Schitoviridae (15.79%), Autographiviridae (9.43%), and Schitoviridae (7.17%); whereas the top four abundant viral compositions in WB were Drexlerviridae (28.88%), Ackermannviridae (23.07%), Adenoviridae (22.16%), and Alloherpesviridae (20.41%). Stamp differential analysis showed significant differences between DP and WB for 11 viruses (family level) (Figure 4C), including Ackermannviridae, Autographiviridae, Autolykiviridae, Baculoviridae, Chaseviridae, Demerecviridae, Iridoviridae, Microviridae, Poxviridae, Schitoviridae, and Zobellviridae. All these viruses exhibited significantly higher relative abundance in DP than WB, particularly Chaseviridae. The Chaseviridae family is lytic phages with double-stranded DNA (dsDNA) that primarily infect bacteria belonging to the Gammaproteobacteria class (Anany et al., 2022). Certain species within Chaseviridae family, such as MLP1, are capable of infecting multidrug-resistant *Escherichia coli* (antibiotic-resistant clinical isolates of uropathogenic *E. coli*) and pathogenic *E. coli* strains found in the gut, including enteroaggregative *E. coli* and diffusely adherent *E. coli* (Vera-Mansilla et al., 2022).

### 3.4 Antibiotic resistance genes composition in DP and WB

**Figure 5:**
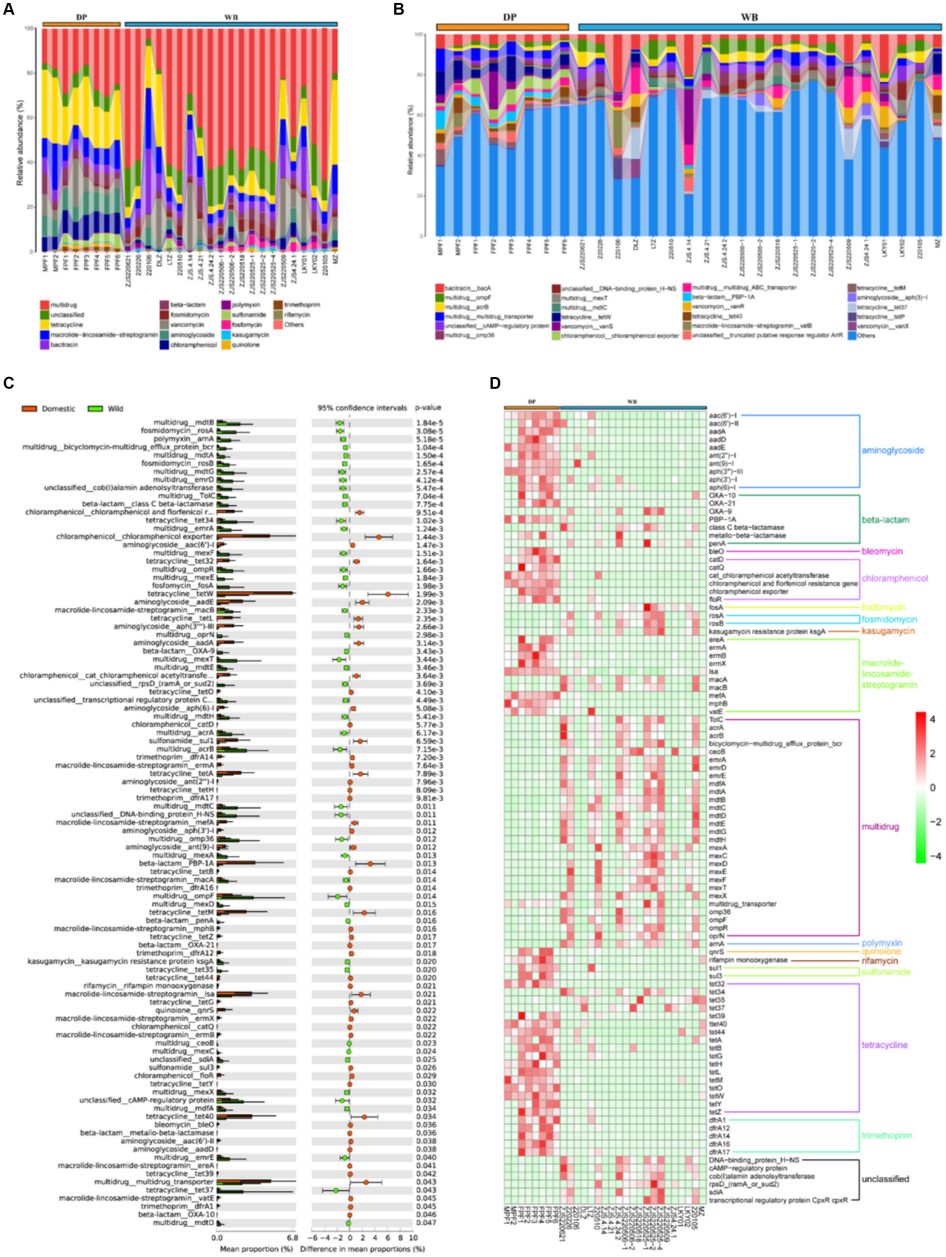
Composition and dissimilarity of ARGs between DP and WB. **(A)** The relative abundance of ARG types in DP and WB; **(B)** The relative abundance of ARG subtypes in DP and WB; **(C)** Significantly different of 99 ARG subtypes between DP and WB; **(D)** Heat map of 99 ARG subtypes with significant differences between DP and WB, red and green represent higher and lower concentrations of metabolites in DP and WB, respectively.

With the development of livestock and poultry farming, the use of antibiotics is common in animal husbandry (Xie et al., 2016; Salerno et al., 2022). The addition of antibiotics can improve survival rates and reduce breeding costs, but their overuse can lead to intestinal microbial imbalance, decreased immune response, and antibiotic resistance (Li et al., 2019). It has been reported that a significant portion of antibiotics added to livestock cannot be fully utilized by the organism. These residual antibiotics may enter other animals or human bodies through the food chain, consequently contaminating water sources and soil, posing a potential threat to the environment and host health (Pal et al., 2016; Qiao et al., 2018; Li et al., 2019). In this study, a total of 20 ARG types containing 254 subtypes were detected in WB and DP. study, a total of 20 ARG types containing 254 subtypes were detected in WB and DP (Figure 5A). Among those, multidrug (24.01%), tetracycline (20.75%), aminoglycoside (8.91%), chloramphenicol (8.80%), macrolide-lincosamide-streptogramin (8.71%), and vancomycin (6.62%) are dominant ARGs types in DP. The pattern of ARGs in DP may be largely associated with antibiotic usage in pig farms. Tetracyclines and penicillins are the two most commonly used antibiotics in global pig production (Lekagul et al., 2019), with other commonly used antibiotics in pig farming including aminoglycosides, macrolides, and chloramphenicol, and so on (Lekagul et al., 2020; Matheson et al., 2022). In WB, multidrug resistance genes (57.47%) were overwhelmingly predominant, followed by unclassified genes (12.66%), tetracycline resistance genes (5.81%), and macrolide-lincosamide-streptogramin resistance genes (4.46%). Previous studies have reported that wild boars are reservoirs and vectors for antibiotic resistant bacteria (ARB), and the tetracycline was the most abundant ARG type in European wild boar feces (Dias et al., 2022). At the subtypes level (Figure 5B), in the WB, the four most abundant subtypes of ARGs were multidrug_ompF (4.64%), bacitracin_bacA (4.06%), multidrug_acrB (3.77%), and unclassified_cAMP-regulatoryprotein (3.29%); while in DP, the most abundant four ARGs subtypes were tetracycline_tetW (6.03%), chloramphenicol_chloramphenicolexporter (5.37%), multidrug_multidrug_transporter (4.80%), and vancomycin_vanS (4.23%).

Stamp differential analysis revealed significant differences in 99 ARG subtypes between DP and WB (Figure 5C). Specifically, tetracycline_tetW (*p* = 0.001993), chloramphenicol_chloramphenicol exporter (*p* = 0.001441), and beta-lactam_PBP-1A (*p* = 0.013) were more abundant in DP, while tetracycline_tet37 (*p* = 0.043), multidrug_ompF (*p* = 0.014), and multidrug_mexT (*p* = 0.0034) were significantly enriched in WB. As shown in Figure 5D, these 99 distinct subtypes can be mainly categorized into 16 classes of ARG types. Among these types, aminoglycoside, bleomycin, chloramphenicol, quinolone, rifamycin, sulfonamide, trimethoprim, macrolide-lincosamide-streptogramin (including ereA, ermA, ermB, ermX, lsa, mefA, mphB, vatE), and tetracycline (including tet39, tet40, tet44, tetA, tetB, tetG, tetH, tetL, tetM, tetO, tetW, tetY, tetZ) exhibited enrichment in DP; whereas multidrug, polymyxin, fostomycin, fosmidomycin, kasugamycin, macrolide-lincosamide-streptogramin (macA and macB), tetracycline (including tet34, tet35, tet37), and unclassified (including DNA-binding_protein_H-NS, cAMP-regulatory protein, cob(I)alamin adenosyltransferase, rpsD_(ramA_or_sud2), sdiA, transcriptional regulatory protein CpxR cpxR) were more abundant in WB. The previous analysis of ARGs data from wild boar, coyotes, domesticated cattle, and the surrounding environment indicates that wild animals may possess a greater capacity to carry a higher abundance of ARGs or ARBs compared to livestock, potentially serving as a reservoir for the outbreak of antibiotic-resistant microbes (Lee et al., 2022). Importantly, with the increasing likelihood of contact between wild animals and livestock or humans, monitoring and controlling antibiotic resistance becomes crucial to prevent potential epidemic outbreaks and spread. Therefore, it is imperative to strengthen research on the transmission pathways of antibiotic-resistant microorganisms in natural ecosystems to ensure public health and ecosystem integrity.

### 3.5 Human pathogenic bacteria composition differences between DP and WB

**Figure 6:**
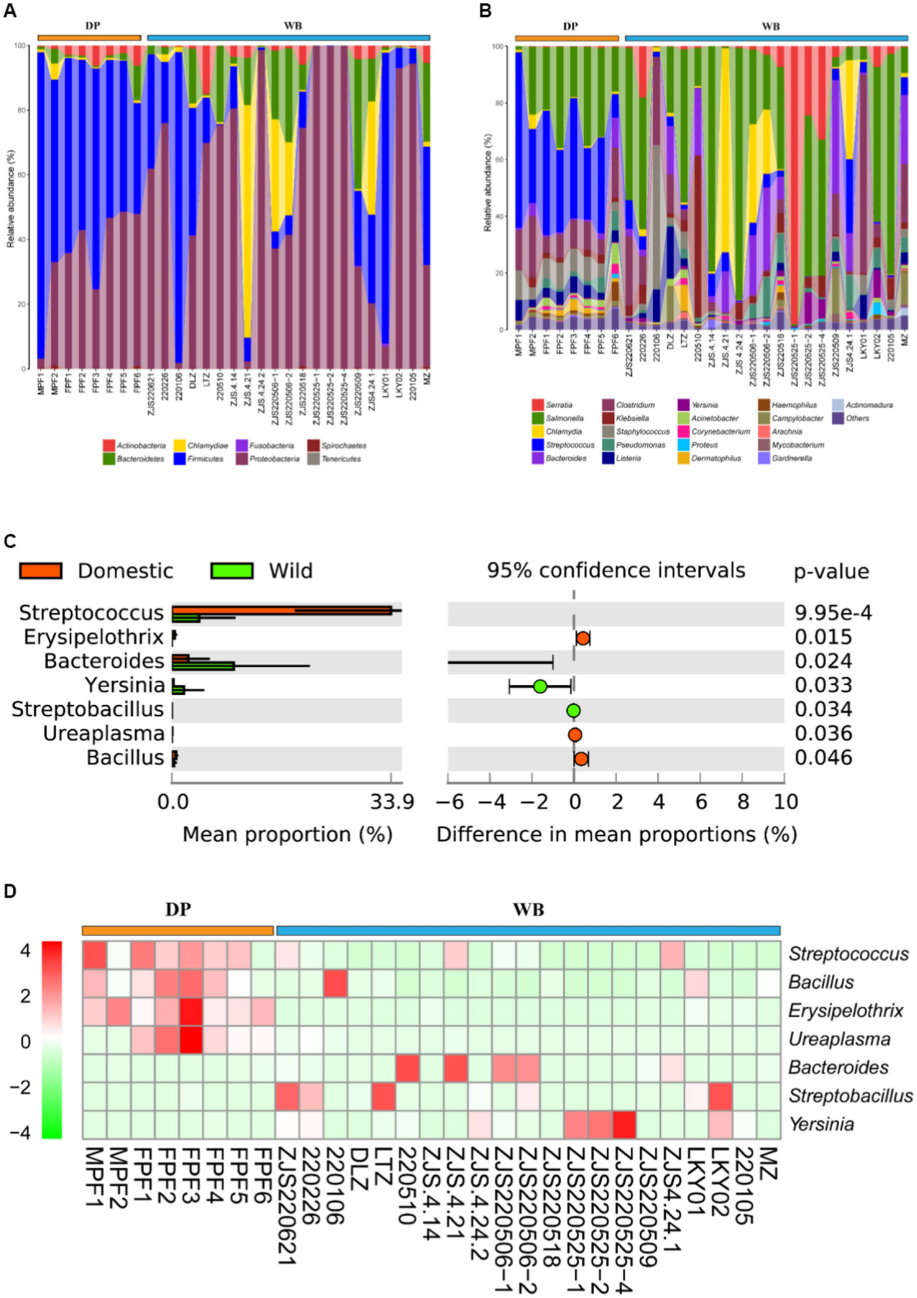
Composition and dissimilarity of HPB between DP and WB. **(A)** The relative abundance of HPB (phylum level) in DP and WB; **(B)** The relative abundance of HPB (genus level) in DP and WB; **(C)** Significantly different of 7 HPB between DP and WB; **(D)** Heat map of 7 HPB subtypes with significant differences between DP and WB, red and green represent higher and lower concentrations of metabolites in DP and WB, respectively.

Animal feces may carry various human pathogenic bacteria (HPB). When the feces of domestic pigs and wild boars are left untreated and directly exposed in the human environment, especially when HPB enter the human body through contaminated water sources or food chains, this poses a potential threat to human health (Penakalapati et al., 2017; Delahoy et al., 2018; Li et al., 2020). Therefore, proper handling and disposal of animal feces are crucial for preventing disease transmission. At the phylum level (Figure 6A), Firmicutes (60.54%) and Proteobacteria (33.73%) were the predominant HPB in DP, while in WB, Proteobacteria (73.13%) dominated, followed by Chlamydiae (10.44%) and Firmicutes (9.71%). At the genus level (Figure 6B), the four most abundant HPB in DP were *Streptococcus* (37.36%), *Salmonella* (23.16%), *Clostridium* (10.07%), and *Staphylococcus* (7.59%), whereas in WB, the prevailing HPB were *Serratia* (42.95%), *Salmonella* (20.33%), *Chlamydia* (10.44%), and *Bacteroides* (5.68%). *Salmonella* was highly represented in both domestic pigs and wild boars in this study. *Salmonella* is one of the most common pathogens causing salmonellosis and other outbreaks of foodborne illness in humans (Mataragas et al., 2008).

Further differential analysis revealed significant differences among 7 HPB between DP and WB (Figure 6C). *Streptococcus* (*p* = 0.000994), *Bacillus* (*p* = 0.024), *Erysipelothrix* (*p* = 0.015), and *Ureaplasma* (*p* = 0.036) significantly enriched in DP. *Streptococcus suis* is commonly recognized an opportunistic pathogen for respiratory infections (Gajdács et al., 2020). Humans can become infected with porcine streptococcus through contact with or consumption of pork or other pig products, leading to severe illnesses such as meningitis, septicemia, streptococcal toxic shock syndrome, endophthalmitis, and arthritis (Camporese et al., 2007; Huong et al., 2014; Gajdács et al., 2020). In DP, *Bacillus cereus* is the most abundant HPB in *Bacillus* genus (Supplementary Table S3). *Bacillus cereus* is an opportunistic pathogen renowned for its significant pathogenicity in foodborne illnesses (Ehling-Schulz et al., 2019). *Bacillus cereus* strains exhibit a wide range of cytotoxic effects from no cytotoxicity to high cytotoxicity in cell culture (Stark et al., 2013; Jeßberger et al., 2015), and it is capable of inducing diverse systemic diseases, such as CNS infections, endocarditis, respiratory and urinary tract complications, wound infections, septicemia, as well as localized wound and ocular infections (Pinna et al., 2001; Bottone, 2010). In livestock, *Streptococcus suis* can induce mammary gland infections in cows and goats (Savini, 2016), as well as result in symptoms such as enteritis and renal failure in domestic pigs (Calvigioni et al., 2022). *Erysipelothrix rhusiopathiae*, the principal pathogen of the genus *Erysipelothrix* in DP (Supplementary Table S3), is a zoonotic pathogen transmitted through aquatic skin infections, causing erysipelas in pig and avian (Wang et al., 2010; Vasagar et al., 2018; Zautner et al., 2022). *Ureaplasma*, the most prevalent genital Mycoplasma isolated from the urogenital tract of humans, is capable of causing urinary and reproductive tract infections, infertility, as well as triggering preterm labor and neonatal disorders, being commonly found in both humans and animals and transmitted through various means such as environmental contact and airborne droplets (Kokkayil and Dhawan, 2015). Human exposure to pig manure containing various HPB is common in livestock farming or household breeding, which may lead to the spread of pathogens in the population and increase the risk of disease. Therefore, it is important to take preventive measures to reduce health risks when humans come into contact with pigs and their environments.

In contrast, *Bacteroides* (*p* = 0.024), *Streptobacillus* (*p* = 0.034), and *Yersinia* (*p* = 0.033) were more abundant in WB (see Figure 6D). In comparison to Bacteroides and Streptobacillus, *Yersinia* has been more commonly reported in domestic pigs and wild boars. Enteropathogenic *Yersinia* genus is also commonly detected in wildlife including wild boars (Sannö et al., 2023). *Yersinia pestis*, *Yersinia enterocolitica*, and *Yersinia pseudotuberculosis* were identified as the major HPB of the genus *Yersinia* in wild boars in this study (Supplementary Table S3). These three HPB represent the *Yersinia* genus’s pathogenicity toward humans (Chlebicz and Śliżewska, 2018; Augustyniak and Pomorska-Mól, 2023). Of these, *Y. pestis* is responsible for the highly lethal plague disease, while *Y. enterocolitica* and *Y. pseudotuberculosis* contribute to yersiniosis (Gupta et al., 2020; Augustyniak and Pomorska-Mól, 2023). Pigs serve as the primary reservoir for human pathogenic *Yersinia enterocolitica* (Råsbäck et al., 2018). While Streptobacillus typically transmitted through rat bites, leads to an infectious disease known as rat-bite fever (Elliott, 2007; Zhang et al., 2019).

## 4 Conclusion

In conclusion, this study reveals significant differences in gut microbial composition, metabolic profiles, virome composition, ARGs, and HPB between domestic pigs (DP) and wild boars (WB) in urban environments. DP showed a higher Firmicutes/Bacteroidetes ratio and enrichment of bacterial genera linked to domestication and modern feeding practices. Metabolomic analysis identified differential metabolite profiles, especially in the pantothenate and CoA biosynthesis pathway, highlighting dietary influences. DP also exhibited a distinct gut virome, enriched with lytic phages like Chaseviridae. Variations in ARGs and HPB composition suggest potential health risks from pig feces contact. Future research should focus on understanding the mechanisms driving these differences and their implications for host health. Additionally, strategies to mitigate public health risks related to urban wildlife and domestic animals should be developed based on these findings.

## Data availability statement

The data presented in the study are deposited in the NCBI repository, accession number PRJNA1104418.

## Ethics statement

Ethical approval was not required for the studies involving animals in accordance with the local legislation and institutional requirements because this study adhered to animal ethical principles and was deemed exempt from ethical review. Written informed consent was obtained from the owners for the participation of their animals in this study.

## Author contributions

JD: Writing – original draft, Methodology, Investigation, Formal analysis, Data curation, Conceptualization. XC: Writing – original draft, Formal analysis. XW: Writing – original draft, Investigation. FZ: Writing – original draft, Investigation. LW: Writing – original draft, Methodology, Conceptualization. LZ: Writing – review & editing, Methodology, Formal analysis, Conceptualization.
