# Supplementary material for: Multi-omics analysis of gut microbiota and metabolites reveals contrasting profiles in domestic pigs and wild boars across urban environments
Source: Front Microbiol. 2024 Aug 13;15:1450306. doi: 10.3389/fmicb.2024.1450306 (PMC11347354; doi:10.3389/fmicb.2024.1450306)
Supplement: Supplementary file 1 [file Table_1.docx]

# Supplementary Information

Table S1 Information on the samples of domestic Pigs (DP) and wild Boars (WB) utilized across different analyses in this study. “Yse” denotes the sample's inclusion in the analysis, “No” indicates exclusion.

| **Group** | **ID Name** | **Sampling site** | **Full-length 16S Analysis** | **Metagenomics Analysis** | **Metabolomics Analysis** |
| --- | --- | --- | --- | --- | --- |
| DP | MPF1 | Pukou District (Nanjing, Jiangsu Province, China) | Yes | Yes | Yes |
| DP | MPF2 | Pukou District (Nanjing, Jiangsu Province, China) | Yes | Yes | Yes |
| DP | FPF1 | Jiangning District (Nanjing, Jiangsu Province, China) | Yes | Yes | Yes |
| DP | FPF2 | Jiangning District (Nanjing, Jiangsu Province, China) | Yes | Yes | Yes |
| DP | FPF3 | Jiangning District (Nanjing, Jiangsu Province, China) | Yes | Yes | Yes |
| DP | FPF4 | Jiangning District (Nanjing, Jiangsu Province, China) | Yes | Yes | Yes |
| DP | FPF5 | Jiangning District (Nanjing, Jiangsu Province, China) | Yes | Yes | Yes |
| DP | FPF6 | Jiangning District (Nanjing, Jiangsu Province, China) | Yes | Yes | Yes |
| WB | ZJS220621 | Xuanwu District (Nanjing, Jiangsu Province, China) | Yes | Yes | Yes |
| WB | 220226 | Jiangning District (Nanjing, Jiangsu Province, China) | Yes | Yes | Yes |
| WB | LKY01 | Jiangning District (Nanjing, Jiangsu Province, China) | Yes | Yes | Yes |
| WB | LKY02 | Jiangning District (Nanjing, Jiangsu Province, China) | Yes | Yes | Yes |
| WB | MZ | Jiangning District (Nanjing, Jiangsu Province, China) | Yes | Yes | Yes |
| WB | 220105 | Pukou District (Nanjing, Jiangsu Province, China) | Yes | Yes | Yes |
| WB | 220106 | Pukou District (Nanjing, Jiangsu Province, China) | Yes | Yes | Yes |
| WB | DLZ | Pukou District (Nanjing, Jiangsu Province, China) | Yes | Yes | Yes |
| WB | LTZ | Pukou District (Nanjing, Jiangsu Province, China) | Yes | Yes | Yes |
| WB | 220510 | Xuanwu District (Nanjing, Jiangsu Province, China) | Yes | Yes | Yes |
| WB | ZJS.4.14 | Xuanwu District (Nanjing, Jiangsu Province, China) | Yes | Yes | Yes |
| WB | ZJS.4.21 | Xuanwu District (Nanjing, Jiangsu Province, China) | Yes | Yes | Yes |
| WB | ZJS.4.24.2 | Xuanwu District (Nanjing, Jiangsu Province, China) | Yes | Yes | Yes |
| WB | ZJS220506-1 | Xuanwu District (Nanjing, Jiangsu Province, China) | Yes | Yes | Yes |
| WB | ZJS220506-2 | Xuanwu District (Nanjing, Jiangsu Province, China) | Yes | Yes | Yes |
| WB | ZJS220507 | Xuanwu District (Nanjing, Jiangsu Province, China) | Yes | No | No |
| WB | ZJS220518 | Xuanwu District (Nanjing, Jiangsu Province, China) | Yes | Yes | Yes |
| WB | ZJS22022 | Xuanwu District (Nanjing, Jiangsu Province, China) | Yes | No | No |
| WB | ZJS220524 | Xuanwu District (Nanjing, Jiangsu Province, China) | Yes | No | No |
| WB | ZJS220525-1 | Xuanwu District (Nanjing, Jiangsu Province, China) | Yes | Yes | Yes |
| WB | ZJS220525-2 | Xuanwu District (Nanjing, Jiangsu Province, China) | Yes | Yes | Yes |
| WB | ZJS220525-3 | Xuanwu District (Nanjing, Jiangsu Province, China) | Yes | No | No |
| WB | ZJS220525-4 | Xuanwu District (Nanjing, Jiangsu Province, China) | Yes | Yes | Yes |
| WB | ZJS220525-5 | Xuanwu District (Nanjing, Jiangsu Province, China) | Yes | No | No |
| WB | ZJS220526-1 | Xuanwu District (Nanjing, Jiangsu Province, China) | Yes | No | No |
| WB | ZJS220526-2 | Xuanwu District (Nanjing, Jiangsu Province, China) | Yes | No | No |
| WB | ZJS220509 | Xuanwu District (Nanjing, Jiangsu Province, China) | Yes | Yes | Yes |
| WB | ZJS4.24.1 | Xuanwu District (Nanjing, Jiangsu Province, China) | Yes | Yes | Yes |

Table S2 41 Significantly different metabolites in domestic pigs (DP) and wild boars (WB).

| **Metabolite name** | **Mean**  **(WB)** | **Mean**  **(DP)** | **Fold**  **Change** | **VIP**  **Value** | **P**  **Value** |
| --- | --- | --- | --- | --- | --- |
| (3R, 6'Z)-3,4-Dihydro-8-hydroxy-3-(6-pentadecenyl)-1H-2-benzopyran-1-one | 0.000101116 | 0.00262137 | 0.038573687 | 1.831972979 | 0.000362124 |
| (Cyclohexylmethyl)pyrazine | 0.000475398 | 0.00087561 | 0.542933326 | 1.128330104 | 0.019822821 |
| 10E,12Z-Octadecadienoic acid | 0.010502373 | 0.04677134 | 0.224547181 | 1.200753373 | 8.34555E-06 |
| 2,5-Dihydro-2,4-dimethyloxazole | 0.000406162 | 0.001222381 | 0.332271081 | 1.377271549 | 0.008787231 |
| 2-acetyl-1-alkyl-sn-glycero-3-phosphocholine | 0.005515736 | 0.000581259 | 9.489298551 | 1.108539763 | 0.014607631 |
| 3-Aminobutanoic acid | 0.000742212 | 0.004115027 | 0.180366185 | 1.912906024 | 2.39818E-07 |
| 3-Carboxy-4-methyl-5-propyl-2-furanpropionic acid | 0.006392118 | 0.014506359 | 0.440642489 | 1.164519549 | 0.03140706 |
| 4-Acetylbutyrate | 0.016974281 | 0.106636793 | 0.159178469 | 1.544814719 | 0.037782857 |
| 4-Trimethylammoniobutanoic acid | 8.02937E-05 | 0.000533699 | 0.150447443 | 1.651045991 | 0.049667543 |
| 5-Methylcytosine | 0.000275991 | 0.000867139 | 0.318277885 | 1.502064834 | 0.013028592 |
| Acetylleucine | 0.001971663 | 0.000153633 | 12.83362732 | 1.063534613 | 0.005136344 |
| Adenine | 0.002762722 | 0.028124761 | 0.098230943 | 1.849456236 | 0.016222504 |
| Alanyl-Leucine | 0.000382091 | 0.003159189 | 0.120945785 | 1.512792455 | 0.004437956 |
| Betaine | 0.024202331 | 0.145818004 | 0.165976288 | 1.556227836 | 0.005470419 |
| Denudatine | 0.000708083 | 0.002210187 | 0.320372479 | 1.025570831 | 0.000185007 |
| Dodecanoic acid | 0.001568933 | 0.049791906 | 0.031509792 | 1.751562566 | 0.000508429 |
| D-Pantothenic acid | 0.008418541 | 0.000209537 | 40.17694296 | 1.043308174 | 0.000322125 |
| Gingerol | 0.036104399 | 0.002856608 | 12.63890613 | 1.266739259 | 0.002864213 |
| Histamine | 0.000204555 | 0.00191361 | 0.106894612 | 1.447861873 | 0.000621717 |
| H-LEU-VAL-OH | 0.004007819 | 0.01862511 | 0.215183661 | 1.272377988 | 0.000589118 |
| L-Alanine | 0.000486221 | 0.000989855 | 0.491203946 | 1.00586057 | 0.048919708 |
| Lauroyl diethanolamide | 0.007740691 | 7.29093E-05 | 106.1687331 | 1.963278179 | 0.000242605 |
| L-Carnitine | 0.00328169 | 0.015985496 | 0.205291698 | 1.614243267 | 0.008331311 |
| Leucyl-Isoleucine | 0.003828226 | 0.010051524 | 0.380860254 | 1.053962075 | 0.024275072 |
| L-Hexanoylcarnitine | 0.000415374 | 0.002016523 | 0.20598519 | 1.623034401 | 0.04134219 |
| L-Valine | 0.026469286 | 0.000689468 | 38.39086543 | 1.510593965 | 0.000606312 |
| LysoPC(18:3(6Z,9Z,12Z)) | 0.000550368 | 7.12689E-05 | 7.722408025 | 1.052641232 | 0.001388032 |
| LysoPE(18:1(9Z)/0:0) | 0.000470841 | 0.003697228 | 0.127349813 | 1.546748584 | 0.020039399 |
| Malonic acid | 0.002999723 | 0.010969746 | 0.273454214 | 1.409033789 | 0.008191822 |
| Nicotinamide N-oxide | 0.000316487 | 7.06785E-05 | 4.477841807 | 1.234114687 | 0.002936467 |
| Palmitic acid | 0.025298351 | 0.006872952 | 3.680856668 | 1.07157141 | 0.005606588 |
| PC(18:1(11Z)/14:0) | 0.005228791 | 0.000162034 | 32.26974083 | 1.40955759 | 0.011111388 |
| PC(20:1(11Z)/14:0) | 0.041727013 | 0.000248122 | 168.1711248 | 1.591381218 | 0.010619007 |
| Phthalic acid | 0.002743861 | 0.000432162 | 6.349152329 | 1.407671392 | 0.001776348 |
| Propionic acid | 0.001798451 | 0.011291631 | 0.159272923 | 1.681798097 | 0.032479823 |
| Pyrrolidine | 0.008966526 | 0.00034754 | 25.80000728 | 1.703853535 | 0.003875102 |
| Riboflavin | 0.000140171 | 0.000603566 | 0.232237458 | 1.373705972 | 0.008823695 |
| Stearoylcarnitine | 0.001042246 | 1.63072E-05 | 63.91319094 | 2.207954181 | 0.021762269 |
| Syringaldehyde | 0.000234721 | 0.000562098 | 0.417581198 | 1.374635998 | 0.029396453 |
| Triethanolamine | 0.051864659 | 0.000169284 | 306.3762335 | 1.121889659 | 0.004743333 |
| Uracil | 0.033532301 | 0.000232809 | 144.033338 | 1.21389036 | 0.004406618 |

Table S3 Relative abundance of composition of human pathogenic bacteria (HPB; species level) in domestic pigs (DP) and wild boars (WB).

| **HPB Species** | **DP** | **WB** |
| --- | --- | --- |
| *Salmonella enterica* | 23.16% | 20.33% |
| *Streptococcus pyogenes* | 19.33% | 0.31% |
| *Streptococcus suis* | 16.53% | 2.16% |
| *Staphylococcus aureus* | 6.45% | 2.01% |
| *Clostridium botulinum* | 4.73% | 1.67% |
| *Listeria monocytogenes* | 4.42% | 0.75% |
| *Clostridium perfringens* | 3.63% | 2.09% |
| *Pseudomonas aeruginosa* | 2.30% | 1.59% |
| *Acinetobacter baumannii* | 1.86% | 0.26% |
| *Clostridium tetani* | 1.67% | 0.13% |
| *Klebsiella oxytoca* | 1.64% | 5.12% |
| *Dermatophilus congolensis* | 1.55% | 0.18% |
| *Bacteroides fragilis* | 1.55% | 5.68% |
| *Streptococcus pneumoniae* | 1.51% | 0.24% |
| *Staphylococcus epidermidis* | 1.14% | 0.22% |
| *Haemophilus influenzae* | 1.00% | 0.21% |
| *Chlamydia trachomatis* | 0.82% | 10.44% |
| *Corynebacterium diphtheriae* | 0.75% | 0.28% |
| *Bacillus cereus* | 0.67% | 0.07% |
| *Campylobacter coli* | 0.49% | 0.16% |
| *Aeromonas hydrophila* | 0.47% | 0.03% |
| *Campylobacter jejuni* | 0.42% | 0.03% |
| *Erysipelothrix rhusiopathiae* | 0.36% | 0.00% |
| *Vibrio cholerae* | 0.22% | 0.04% |
| *Proteus mirabilis* | 0.22% | 0.40% |
| *Edwardsiella tarda* | 0.21% | 0.21% |
| *Gardnerella vaginalis* | 0.19% | 0.05% |
| *Mycobacterium tuberculosis* | 0.18% | 0.06% |
| *Burkholderia mallei* | 0.18% | 0.17% |
| *Bordetella parapertussis* | 0.17% | 0.00% |
| *Treponema vincentii* | 0.16% | 0.02% |
| *Neisseria meningitidis* | 0.15% | 0.01% |
| *Serratia marcescens* | 0.15% | 5.09% |
| *Yersinia pestis* | 0.14% | 0.72% |
| *Morganella morganii* | 0.13% | 0.08% |
| *Providencia rettgeri* | 0.11% | 0.03% |
| *Arachnia propionica* | 0.10% | 0.12% |
| *Yersinia enterocolitica* | 0.10% | 0.41% |
| *Serratia liquefaciens* | 0.09% | 37.86% |
| *Neisseria gonorrhoeae* | 0.08% | 0.09% |
| *Vibrio vulnificus* | 0.08% | 0.01% |
| *Bordetella bronchiseptica* | 0.07% | 0.00% |
| *Providencia alcalifaciens* | 0.07% | 0.07% |
| *Nocardia asteroides* | 0.07% | 0.00% |
| *Plesiomonas shigelloides* | 0.07% | 0.09% |
| *Ureaplasma urealyticum* | 0.06% | 0.00% |
| *Fusobacterium necrophorum* | 0.06% | 0.03% |
| *Corynebacterium pseudotuberculosis* | 0.06% | 0.00% |
| *Campylobacter fetus* | 0.04% | 0.00% |
| *Peptostreptococcus anaerobius* | 0.04% | 0.01% |
| *Bacillus anthracis* | 0.04% | 0.01% |
| *Actinomyces naeslundii* | 0.03% | 0.02% |
| *Proteus penneri* | 0.03% | 0.00% |
| *Yersinia pseudotuberculosis* | 0.02% | 0.08% |
| *Clostridium novyi* | 0.02% | 0.01% |
| *Nocardia farcinica* | 0.02% | 0.01% |
| *Nocardia carnea* | 0.02% | 0.02% |
| *Arcanobacterium haemolyticum* | 0.02% | 0.19% |
| *Clostridium haemolyticum* | 0.02% | 0.00% |
| *Nocardia nova* | 0.01% | 0.00% |
| *Bordetella pertussis* | 0.01% | 0.00% |
| *Nocardia brasiliensis* | 0.01% | 0.00% |
| *Mycobacterium kansasii* | 0.01% | 0.01% |
| *Corynebacterium minutissimum* | 0.01% | 0.00% |
| *Corynebacterium ulcerans* | 0.01% | 0.03% |
| *Pasteurella multocida* | 0.01% | 0.01% |
| *Legionella pneumophila* | 0.01% | 0.01% |
| *Actinomyces israelii* | 0.00% | 0.01% |
| *Proteus vulgaris* | 0.00% | 0.00% |
| *Helicobacter pylori* | 0.00% | 0.00% |
| *Actinomadura pelletieri* | 0.00% | 0.00% |
| *Corynebacterium bovis* | 0.00% | 0.00% |
| *Francisella tularensis* | 0.00% | 0.00% |
| *Actinomadura madurae* | 0.00% | 0.01% |
| *Streptobacillus moniliformis* | 0.00% | 0.01% |
| *Mycobacterium xenopi* | 0.00% | 0.00% |
| *Actinomyces bovis* | 0.00% | 0.00% |
| *Nocardia otitidiscaviarum* | 0.00% | 0.00% |
| *Listeria ivanovii* | 0.00% | 0.00% |
| *Actinomyces gerencseriae* | 0.00% | 0.00% |
| *Kingella kingae* | 0.00% | 0.00% |
| *Eikenella corrodens* | 0.00% | 0.00% |
| *Mycobacterium asiaticum* | 0.00% | 0.00% |
| *Mycobacterium simiae* | 0.00% | 0.00% |
| *Nocardia transvalensis* | 0.00% | 0.00% |

Figure S1 Metabolite identification results of DP and WB. A, Metabolites are divided into 6 super classes; B Heat map of 164 identified metabolites, red and blue represent higher and lower concentrations of metabolites in DP and WB, respectively.


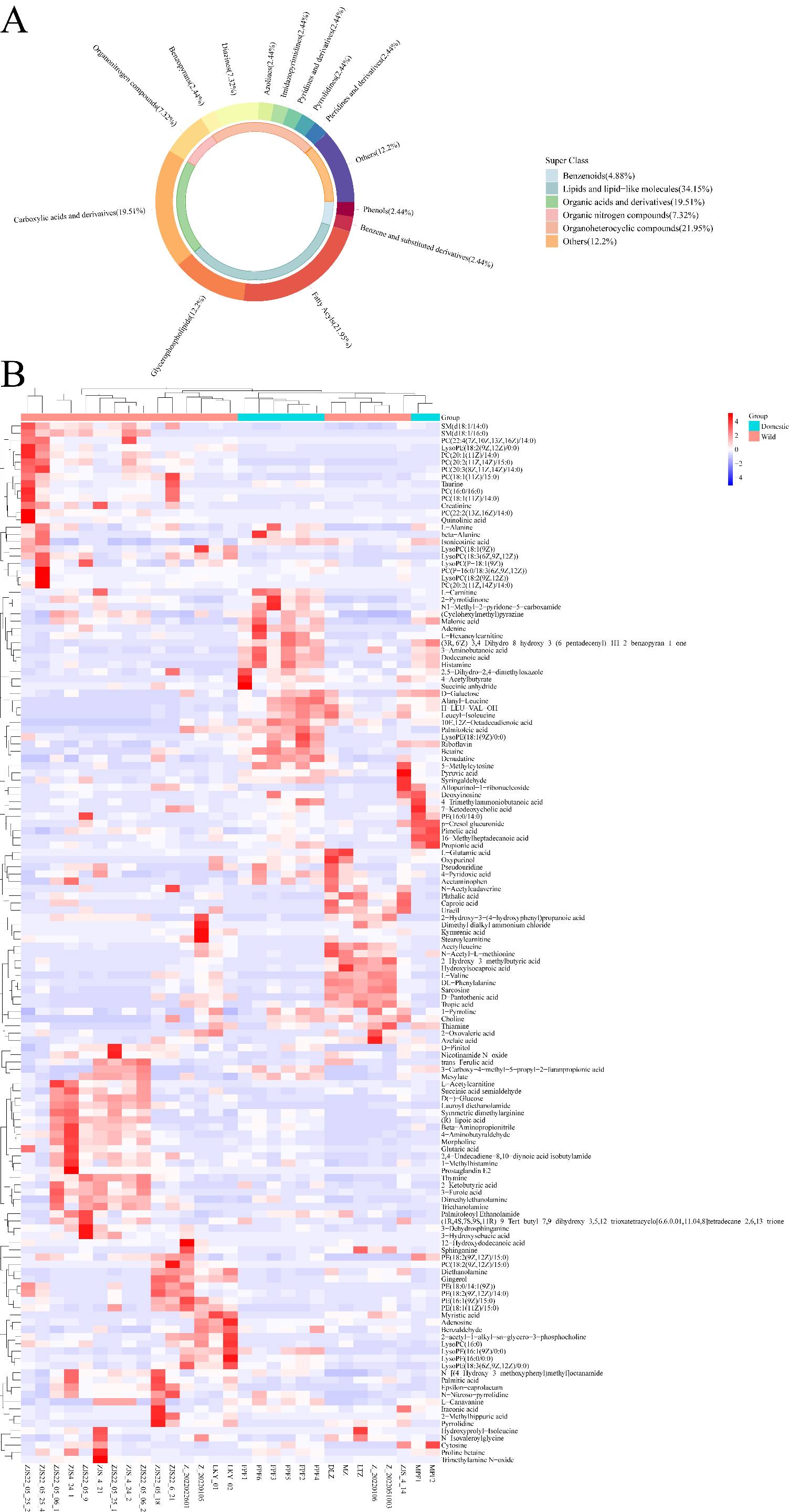


Figure S2 The KEGG pathway diagram of Pantothenate and CoA biosynthesis. Nodes represent metabolites, with red color indicating significantly upregulated differential metabolites.


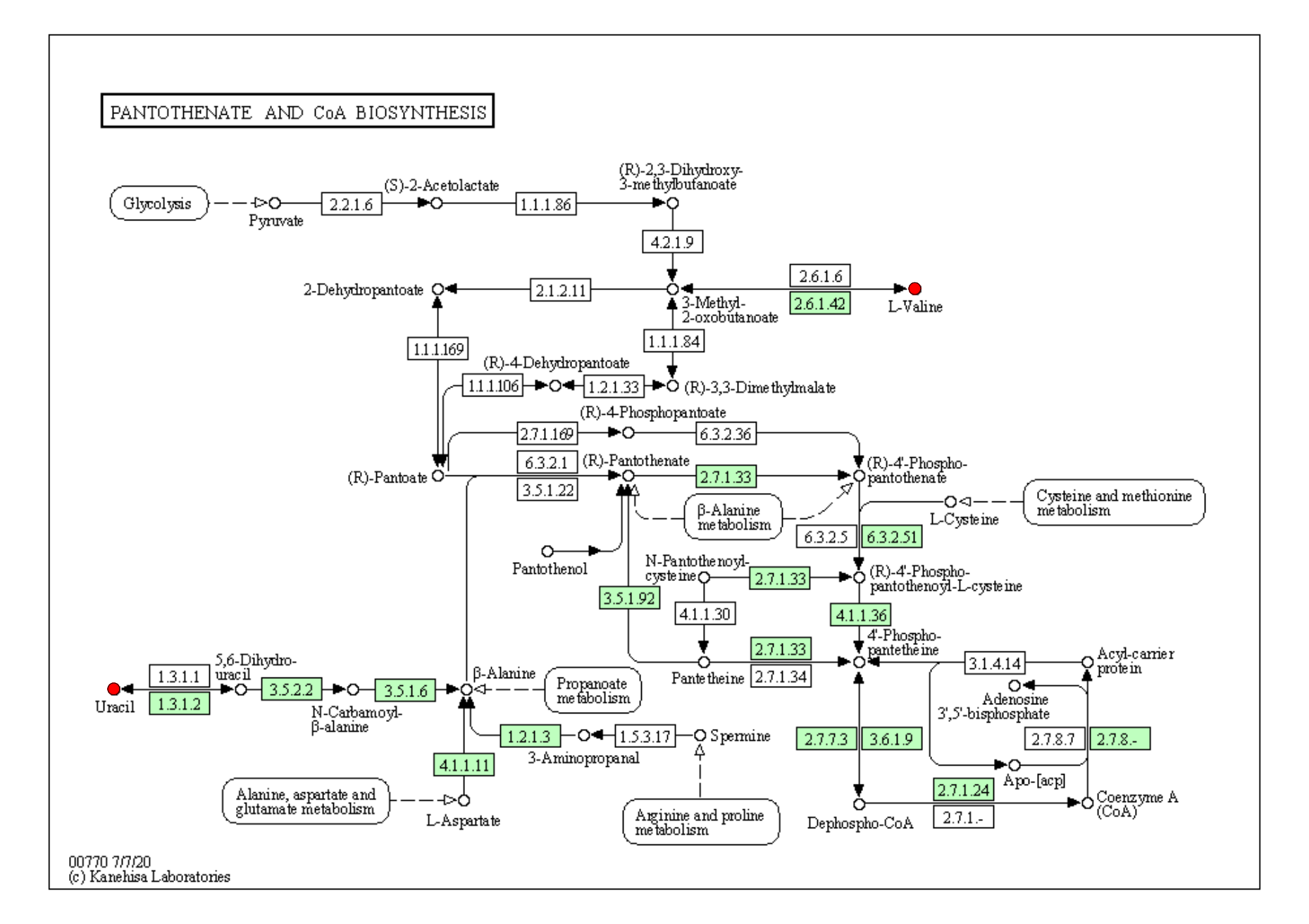


Figure S3 Significantly different status of L-Valine (A) and Uracil (B) between domestic pigs (DP) and wild boars (WB), and their ROC (Receiver Operating Characteristic) curve.


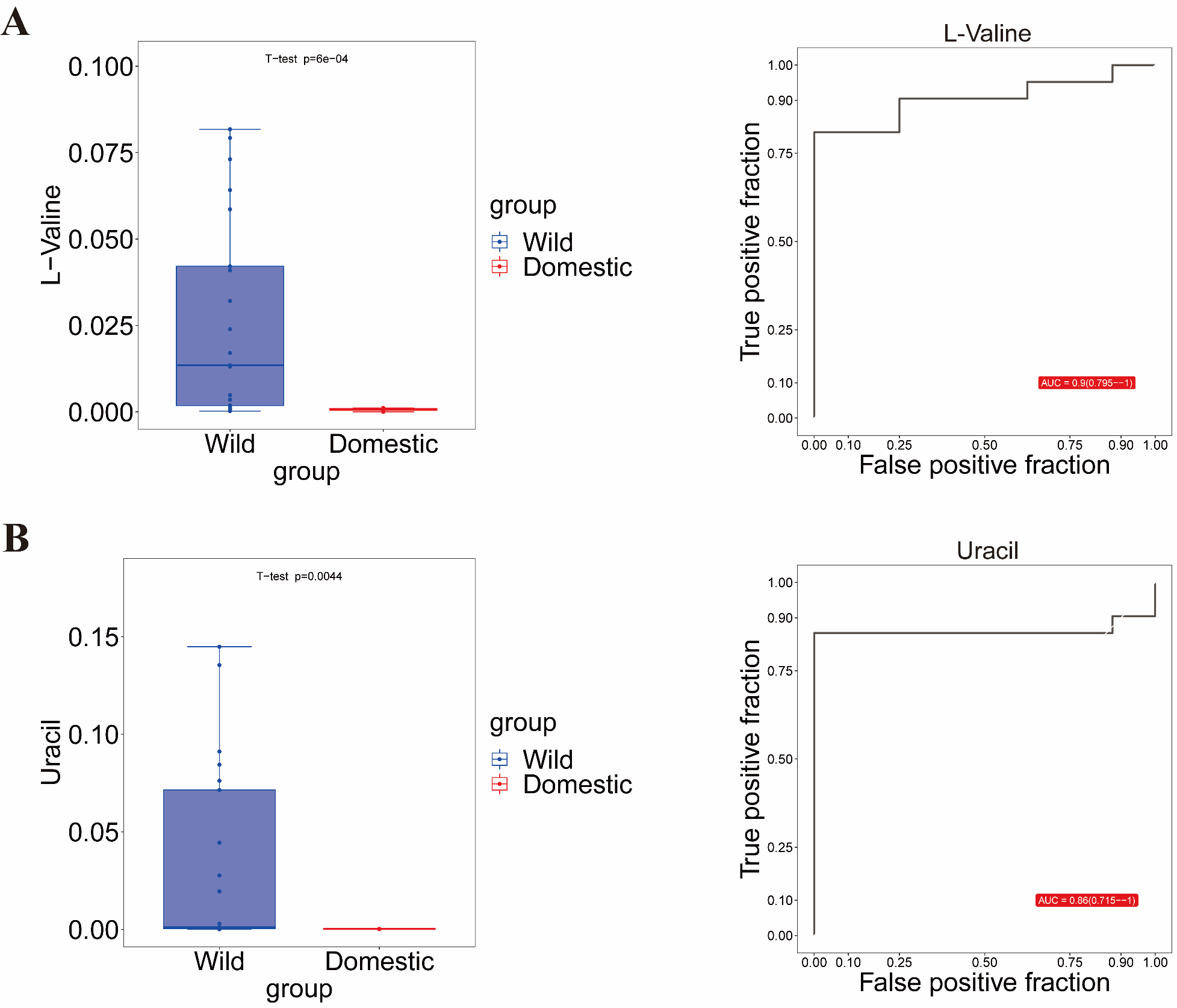


**Metabolome experimental protocol:**

The fecal sample weighing 25 mg was meticulously transferred into an EP tube, followed by the addition of 500 μL of an extraction solution comprising methanol, acetonitrile, and water in a volumetric ratio of 2:2:1, supplemented with an isotopically-labelled internal standard mixture. Subsequently, the samples underwent homogenization at 35 Hz for 4 minutes and were then sonicated for 5 minutes in an ice-water bath. This iterative process of homogenization and sonication was repeated thrice to ensure comprehensive mixing. The samples were subsequently subjected to a 1-hour incubation period at -40 ℃, followed by centrifugation at 12,000 rpm (RCF=13800×g, R=8.6 cm) for 15 minutes at 4 ℃. The resulting supernatant was carefully decanted into fresh glass vials for subsequent analysis. Quality control (QC) samples were meticulously prepared by pooling an equal volume of supernatant from each individual sample.

For LC-MS/MS analyses, a Vanquish UHPLC system (Thermo Fisher Scientific) was employed in conjunction with an Orbitrap Exploris 120 mass spectrometer (Thermo), which was equipped with a UPLC BEH Amide column (2.1 mm × 100 mm, 1.7 μm). The mobile phase composition comprised 25 mM ammonium acetate and 25 mM ammonium hydroxide in water (pH = 9.75) (A), along with acetonitrile (B). The autosampler temperature was rigorously maintained at 4 ℃, and each sample injection volume was precisely set at 2 μL. The Orbitrap Exploris 120 mass spectrometer operated in information-dependent acquisition (IDA) mode, with the ESI source conditions carefully optimized: sheath gas flow rate at 50 Arb, auxiliary gas flow rate at 15 Arb, capillary temperature maintained at 320 ℃, full MS resolution set to 60,000, MS/MS resolution at 15,000, collision energy applied at 10/30/60 in NCE mode, and spray voltage adjusted to 3.8 kV (positive) or -3.4 kV (negative), respectively.

Subsequent to data acquisition, raw datasets underwent conversion to the mzXML format employing ProteoWizard, followed by meticulous processing using an in-house software program developed in R and based on XCMS. This software facilitated peak detection, extraction, alignment, and integration tasks. Metabolite annotation was conducted utilizing an in-house MS2 database (BiotreeDB), employing a stringent cutoff threshold set at 0.3 for enhanced accuracy and reliability.
